# Supplementary material for: Cu(I)- and Pd(0)-Catalyzed Arylation of Oxadiamines with Fluorinated Halogenobenzenes: Comparison of Efficiency
Source: Molecules. 2020 Feb 28;25(5):1084. doi: 10.3390/molecules25051084 (PMC7179129; doi:10.3390/molecules25051084)
Supplement: Supplementary file 1 [file molecules-25-01084-s001.pdf]

Cu(I)- and Pd(0)-Catalyzed Arylation of Oxadiamines with Fluorinated Halogenobenzenes: Comparison of Efficiency

Maria S. Lyakhovich [1], Alexei D. Averin [1,2,*], Olga K. Grigorova [1], Vitaly A. Roznyatovsky [1], Olga A. Maloshitskaya [1] and Irina P. Beletskaya [1,2]

Supporting Information

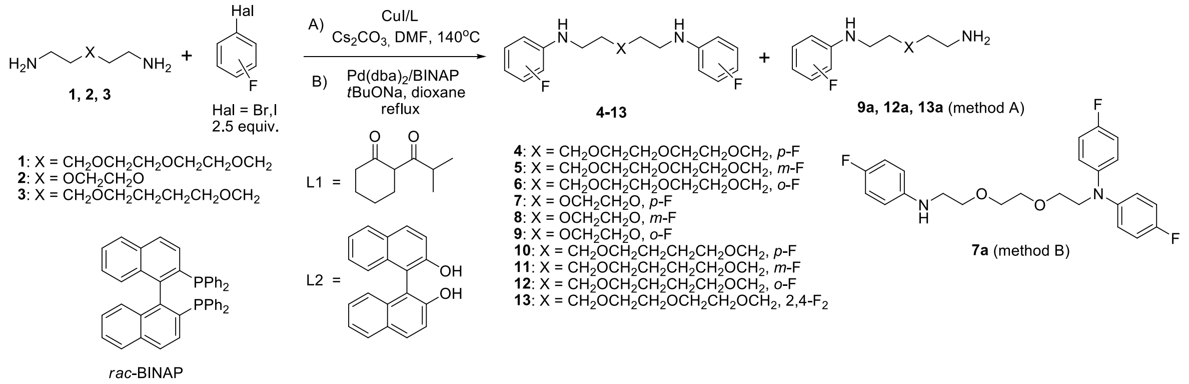

Scheme S1.

Table S1. Cu(I)-catalyzed arylation of oxadiamines **1–3** with fluoroiodo- and bromoflurobenzenes.

Aryl halide	Amine	L (CuI/L, mol%)	Product	Yield, %
4-Fluoroiodobenzene	**1**	L1 (20/40)	**4**	24
4-Fluoroiodobenzene	**1**	L2 (10/20)	**4**	18
4-Bromofluorobenzene	**1**	L1 (20/40)	**4**	35
3-Fluoroiodobenzene	**1**	L1 (20/40)	**5**	82
2-Fluoroiodobenzene	**1**	L1 (20/40)	**6**	77
2-Bromofluorobenzene	**1**	L1 (20/40)	**6**	40[a]
2,4-Difluoroiodobenzene	**1**	L1 (20/40)	**13**	20
			**13a**	19
4-Fluoroiodobenzene	**2**	L1 (20/40)	**7**	34
4-Fluoroiodobenzene	**2**	L2 (10/20)	**7**	62
4-Bromofluorobenzene	**2**	L1 (20/40)	**7**	54
3-Fluoroiodobenzene	**2**	L1 (20/40)	**8**	70
2-Fluoroiodobenzene	**2**	L1 (20/40)	**9**	58
			**9a**	16
2-Bromofluorobenzene	**2**	L1 (20/40)	**9**	52[a]
4-Fluoroiodobenzene	**3**	L1 (20/40)	**10**	58
4-Fluoroiodobenzene	**3**	L2 (10/20)	–	–[b]
4-Bromofluorobenzene	**3**	L1 (20/40)	**10**	48
3-Fluoroiodobenzene	**3**	L1 (20/40)	**11**	77
2-Fluoroiodobenzene	**3**	L1 (20/40)	**12**	30
			**12a**	8
2-Bromofluorobenzene	**3**	L1 (20/40)	**12**	60[a]

[a] Total conversion of NH_2 into NHAr is shown which was estimated from ^1H NMR spectrum of the reaction mixture. [b] Conversion was low, it could not be estimated from ^1H NMR spectrum of the reaction mixture.

Table S2. Pd(0)-catalyzed arylation of oxadiamines **1–3** with bromoflurobenzenes.

Aryl halide	Amine	$Pd(dba)_2$/BINAP, mol%	Product	Yield, %
4-Bromofluorobenzene	**1**	1/1.5	**4**	72
3-Bromofluorobenzene	**1**	1/1.5	**5**	78
2-Bromofluorobenzene	**1**	1/1.5	**6**	74[a)]
2-Bromofluorobenzene	**1**	4/4.5	**6**	98
4-Bromofluorobenzene	**2**	1/1.5	**7**	77[a)]
4-Bromofluorobenzene	**2**	2/2.5	**7**<br>**7a**	73<br>11
3-Bromofluorobenzene	**2**	2/2.5	**8**	98
2-Bromofluorobenzene	**2**	1/1.5	**9**	63
4-Bromofluorobenzene	**3**	1/1.5	**10**	60
3-Bromofluorobenzene	**3**	1/1.5	**11**	73
2-Bromofluorobenzene	**3**	1/1.5	**12**	49

[a)] Total conversion of NH_2 into NHAr is shown which was estimated from ^{1}H NMR spectrum of the reaction mixture.

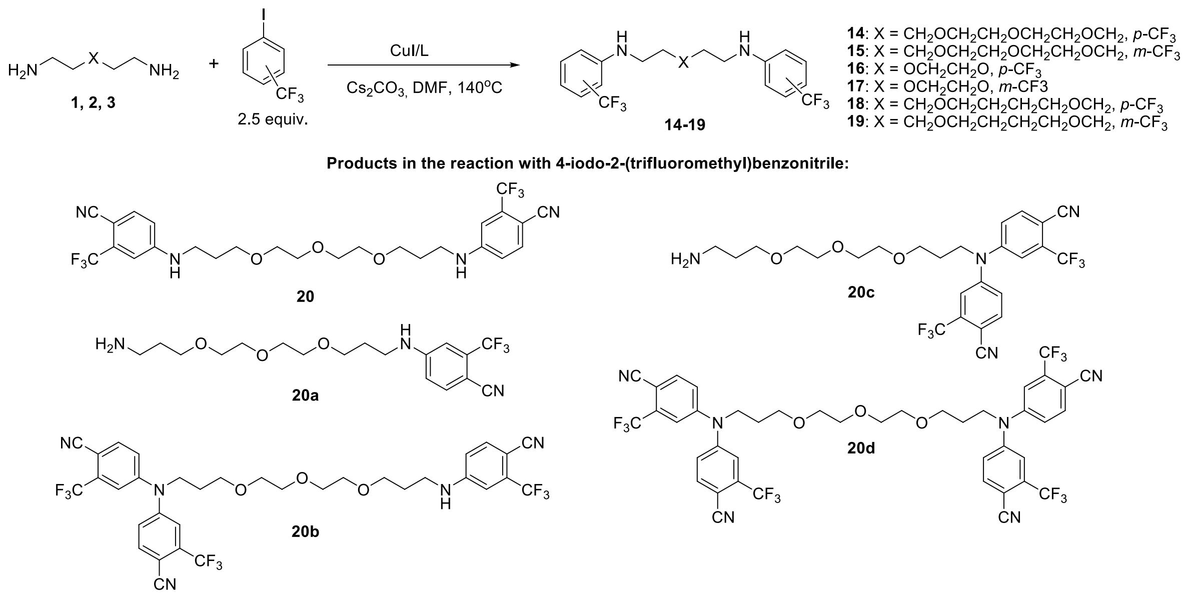

Scheme S2.

Table S3. Cu(I)-catalyzed arylation of oxadiamines **1–3** with iodo(trifluoromethyl)benzenes.

Entry	Aryl halide	Amine	L (CuI/L, mol%)	Product	Yield, %
1	1-Iodo-4-(trifluoromethyl)benzene	**1**	L1 (20/40)	**14**	25
2	1-Iodo-4-(trifluoromethyl)benzene	**1**	L2 (10/20)	**14**	14
3	1-Iodo-3-(trifluoromethyl)benzene	**1**	L1 (20/40)	**15**	21
4	1-Iodo-4-(trifluoromethyl)benzene	**2**	L1 (20/40)	**16**	14
5	1-Iodo-4-(trifluoromethyl)benzene	**2**	L2 (10/20)	—	—
6	1-Iodo-3-(trifluoromethyl)benzene	**2**	L1 (20/40)	**17**	98
7	1-Iodo-4-(trifluoromethyl)benzene	**3**	L1 (20/40)	**18**	28
8	1-Iodo-4-(trifluoromethyl)benzene	**3**	L2 (10/20)	**18**	10
9	1-Iodo-3-(trifluoromethyl)benzene	**3**	L1 (20/40)	**19**	91
10	4-Iodo-2-(trifluoromethyl)benzonitrile	**1**	L1 (20/40)	**20**<br>**20a**<br>**20b**<br>**20c**	17<br>44<br>4<br>12
11	4-Iodo-2-(trifluoromethyl)benzonitrile[a)]	**1**	L1 (20/40)	**20b**<br>**20d**	12<br>9
12	4-Iodo-2-(trifluoromethyl)benzonitrile	**1**	L1 (10/20)	**20**<br>**20b**<br>**20c**	9<br>5<br>5

a) 4 Equiv. of 4-Iodo-2-(trifluoromethyl)benzonitrile were used.

4-((3-(2-(2-(3-Aminopropoxy)ethoxy)ethoxy)propyl)amino)-2-(trifluoromethyl)benzonitrile (**20a**). Obtained as one of several products according to method A from trioxadiamine **1** (0.5 mmol, 110 mg), 4-iodo-2-(trifluoromethyl)benzonitrile (1.25 mmol, 371 mg) in the presence of CuI (19 mg) and 2-isobutyrylcyclohexanone (34 mg). Eluent CH2Cl2–MeOH 20:1. Yield 86 mg (44%). ^{1}H-NMR (400 MHz, $CDCl_3$) δ 1.74 (quintet, 2H, ^{3}J = 6.0 Hz, OCH_2**CH_2**CH_2NH_2), 1.88 (quintet, 2H, ^{3}J = 5.8 Hz, OCH_2**CH_2**CH_2NHAr), 3.27 (q, 2H, ^{3}J = 5.9 Hz, **CH_2**NH_2), 3.36 (q, 2H, ^{3}J = 5.9 Hz, CH_2NAr), 3.54–3.60 (m, 8H, OCH_2), 3.61–3.64 (m, 4H, OCH_2), 5.69 (t, 1H, ^{3}J = 5.0 Hz, NH), 6.65 (dd, 1H, ^{3}J = 8.6 Hz, ^{4}J = 2.0 Hz, H5 (Ph)), 6.82 (d, H, ^{4}J = 2.0 Hz, H2 (Ph)), 7.48 (d, 1H, ^{3}J = 8.6 Hz, H5 (Ph)). NH_2 protons were not unambiguously assigned. ^{13}C-NMR (100.6 MHz, $CDCl_3$) δ 28.2 (1C, OCH_2**CH_2**CH_2NH), 28.3 (1C, OCH_2**CH_2**CH_2NHAr), 36.7 (1C, CH_2NH_2), 41.2 (1C, CH_2NHAr), 69.4 (1C, OCH_2), 69.6 (1C, OCH_2), 70.0 (1C, OCH_2), 70.1 (1C, OCH_2), 70.2 (1C, OCH_2), 70.3 (1C, OCH_2), 94.0 (1C, C4 (Ph)), 110.0 (1C, C6 (Ph)), 113.3 (1C, C2 (Ph)), 118.5 (1C, CN), 122.7 (q, 1C, $^{1}J_{CF}$ = 273.5 Hz, CF_3), 133.8 кв (1C, $^{2}J_{CF}$ = 32.1 Hz, C3 (Ph)), 136.0 (1C, C5 (Ph)), 151.5 (1C, C1 (Ph)). MS (MALDI-TOF+): Calculated for $C_{18}H_{27}F_3N_3O_3$ [*M* + H] 390.2005, found 390.2026.

4,4′-((3-(2-(2-(3-((4-Cyano-3-(trifluoromethyl)phenyl)amino)propoxy)ethoxy)ethoxy)propyl)-azandiyl)bis(2-(trifluoromethyl)benzonitrile) (**20b**). Obtained as one of several products according to method A from trioxadiamine **1** (0.5 mmol, 110 mg), 4-iodo-2-(trifluoromethyl)benzonitrile (2 mmol, 594 mg) in the presence of CuI (19 mg) and 2-isobutyrylcyclohexanone (34 mg). Eluent CH2Cl2–MeOH 200:1. Yield 44 mg (12%). ^{1}H-NMR (400 MHz, $CDCl_3$) δ 1.87–1.94 (m, 4H, OCH_2**CH_2**CH_2N), 3.27 (t, 2H, ^{3}J = 6.0 Hz, CH_2NAr), 3.59–3.62 (m, 8H, OCH_2), 3.65–3.68 (m, 4H, OCH_2), 4.05 (t, 2H, CH_2NAr_2), 5.90 (br. s, 1H, NH), 6.65 (d, 1H, ^{3}J = 8.1 Hz, H6 (Ph)), 6.81 (s, 1H, H2 (Ph)), 7.35 (d, 2H, ^{3}J= 8.0 Hz, ^{4}J = 2.0 Hz, H6 (2Ph)), 7.44 (d, 2H, ^{4}J = 2.2 Hz, H2 (2Ph)), 7.50 (d, 1H, ^{3}J = 8.1 Hz, H5 (Ph)), 7.67 (d, 2H, ^{3}J= 8.0 Hz, H5 (2Ph)).

4,4′-((3-(2-(2-(3-Aminopropoxy)ethoxy)ethoxy)propyl)azanediyl)bis(2-(trifluoromethyl)- benzonitrile) (**20c**). Obtained as one of several products according to method A from trioxadiamine **1** (0.5 mmol, 110 mg), 4-iodo-2-(trifluoromethyl)benzonitrile (2 mmol, 594 mg) in the presence of CuI (19 mg) and 2-isobutyrylcyclohexanone (34 mg). Eluent CH2Cl2–MeOH 50:1. Yield 35 mg (12%). ^{1}H-NMR (400 MHz, $CDCl_3$) δ 1.80 (quintet, 2H, ^{3}J = 5.8 Hz, OCH_2**CH_2**CH_2NH_2), 1.92 (quintet, 2H, ^{3}J = 5.7 Hz, OCH_2**CH_2**CH_2NAr_2), 3.34 (t, 2H, ^{3}J = 5.5 Hz, CH_2NH_2), 3.49 (t, 2H, ^{3}J = 5.7 Hz, CH_2O), 3.56–3.68 (m, 10 H, OCH_2), 4.05 (t, 2H, ^{3}J = 7.0 Hz, CH_2NAr_2), 7.38 (dd, 2H, ^{3}J = 8.6 Hz, ^{4}J = 2.5 Hz, H6 (Ph)), 7.46 (d, 2H, ^{4}J = 2.5 Hz, H2 (Ph)), 7.77 (dd, 2H, ^{3}J = 8.6 Hz, ^{4}J = 2.0 Hz, H5 (Ph)). NH_2 protons were not unambiguously assigned. MS (MALDI-TOF+): Calculated for $C_{26}H_{29}F_6N_4O_3$ [*M* + H] 559.214, found 559.202.

4,4′,4′′,4′′′-((((Oxybis(ethane-2,1-diyl))bis(oxy))bis(propane-3,1-diyl))bis(azanetriyl))tetrakis(2-(trifluoromethyl)benzonitrile) (**20d**). Obtained as one of several products according to method A from trioxadiamine **1** (0.5 mmol, 110 mg), 4-iodo-2-(trifluoromethyl)benzonitrile (2 mmol, 594 mg) in the presence of CuI (19 mg) and 2-isobutyrylcyclohexanone (34 mg). Eluent CH2Cl2–MeOH 200:1. Yield 40 mg (9%). ^{1}H-NMR (400 MHz, $CDCl_3$) δ 1.94 (quintet, 4H, ^{3}J = 5.8 Hz, OCH_2**CH_2**CH_2N), 3.52 (t, 4H, ^{3}J = 5.5 Hz, CH_2O), 3.59–3.62 (m, 4H, OCH_2), 3.68–3.71 (m, 4H, OCH_2), 4.05 (t, 4H, ^{3}J = 6.9 Hz, CH_2N), 7.37 (dd, 4H, ^{3}J = 8.5 Hz, ^{4}J = 2.1 Hz, H6 (Ph)), 7.48 (d, 4H, ^{4}J = 2.1 Hz, H2 (Ph)), 7.76 (dd, 4H, ^{3}J = 8.6 Hz, ^{4}J = 2.1 Hz, H5 (Ph)). ^{13}C-NMR (100.6 MHz, $CDCl_3$) δ 27.2 (2C, OCH_2**CH_2**CH_2NH), 49.2 (2C, CH_2N), 67.2 (2C, CH_2O), 70.3 (2C, CH_2O), 70.4 (2C, CH_2O), 103.0 (4C, C4 (Ph)), 115.5 (4C, CN), 118.7 (q, 4C, $^{3}J_{CF}$ = 4.2 Hz, C2 (Ph)), 122.0 (q, 4C $^{1}J_{CF}$ = 274.0 Hz, CF_3), 123.5 (4C, C6 (Ph)), 134.7(q, 4C, $^{2}J_{CF}$ = 32.2 Hz, C3 (Ph)), 136.4 (4C, C5 (Ph)), 149.4 (4C, C1 (Ph)).

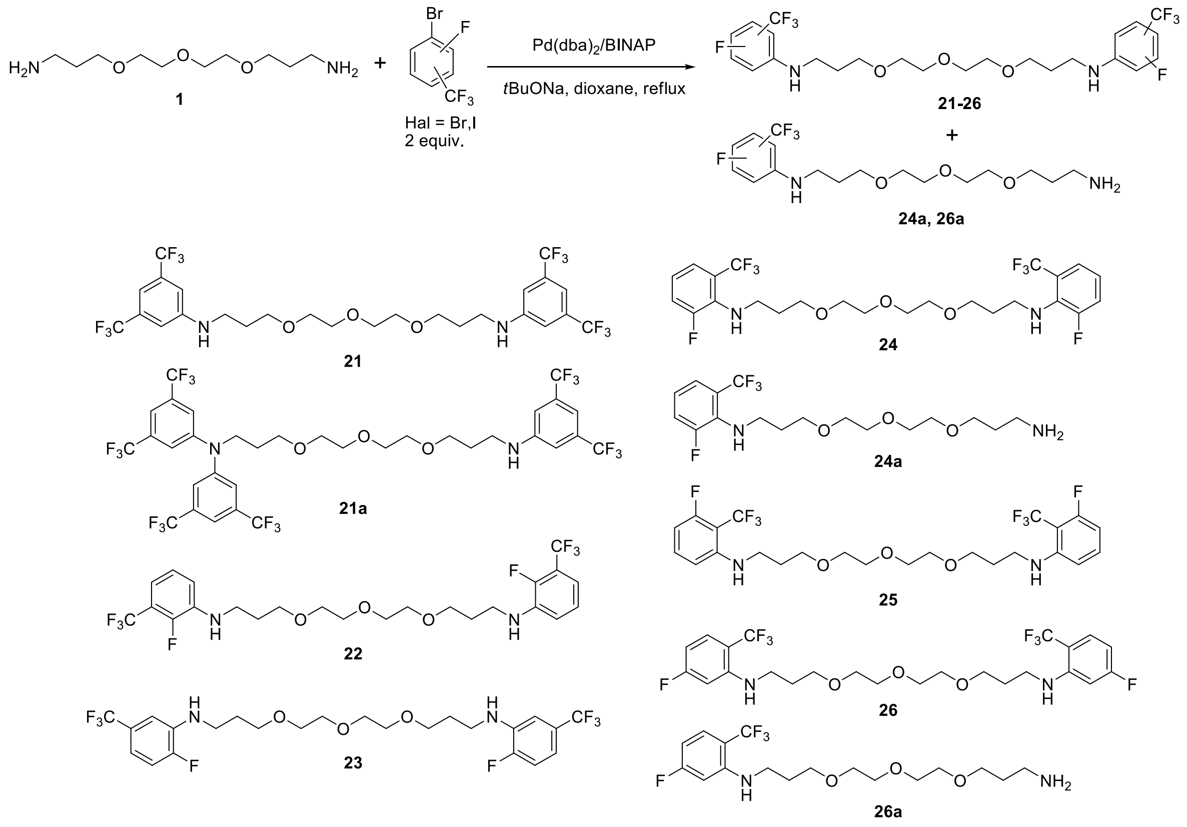

Scheme S3.

Table S4. Pd(0)-catalyzed arylation of trioxadiamine **1** with bromofluro(trifluoromethyl)benzenes.

Entry	Aryl halide	Pd(dba)2/BINAP, mol%	Product	Yield, %
1	1-Bromo-3,5-di(trifluoromethyl)benzene	1/1.5	**21**<br>**21a**	80<br>7
2	3-Bromo-2-fluorobenzotrifluoride	1/1.5	**22**	65
3	3-Bromo-4-fluorobenzotrifluoride	1/1.5	**23**	70
4	2-Bromo-3-fluorobenzotrifluoride	1/1.5	—	—
5	2-Bromo-3-fluorobenzotrifluoride	8/8.5	**24**<br>**24a**	18<br>7
6	2-Bromo-6-fluorobenzotrifluoride	1/1.5	—	—
7	2-Bromo-6-fluorobenzotrifluoride	8/8.5	**25**	17
8	2-Bromo-4-fluorobenzotrifluoride	1/1.5	—	—
9	2-Bromo-4-fluorobenzotrifluoride	4/4.5	**26a**	12
10	2-Bromo-4-fluorobenzotrifluoride	8/8.5	**26**	29

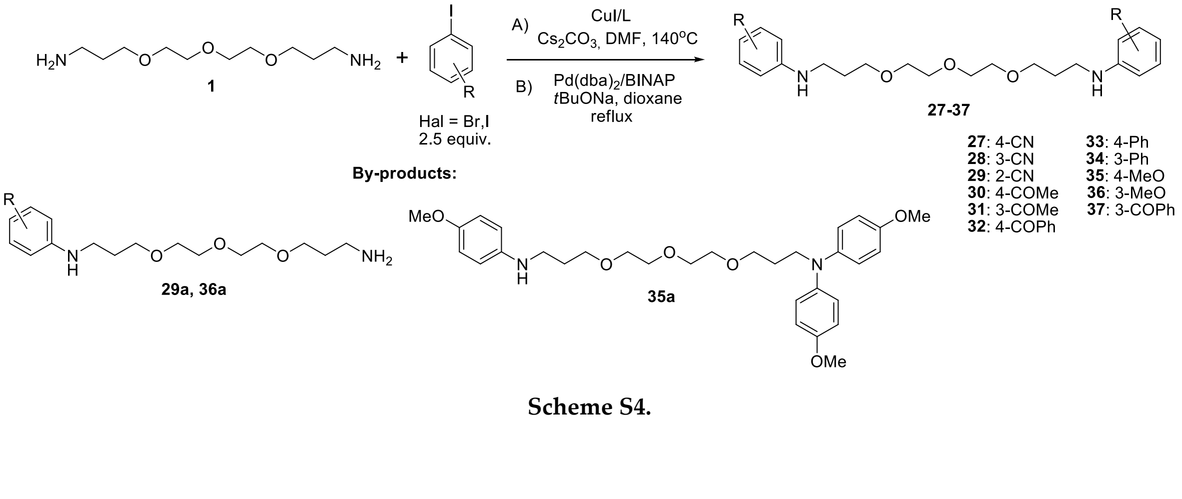

Scheme S4.

Table S5. Cu(I)-catalyzed arylation of trioxadiamine **1** with various aryl iodides (CuI/L1, 20/40 mol%).

Entry	Aryl halide	Product	Yield, %
1	4-Iodobenzonitrile	**27**	91
2	3-Iodobenzonitrile	**28**	78
3	2-Iodobenzonitrile	**29**<br>**29a**	40<br>38
4	4-Iodoacetophenone	**30**	85
5	3-Iodoacetophenone	**31**	45
6	4-Iodobenzophenone	**32**	71
7	4-Iodobiphenyl	**33**	65
8	3-Iodobiphenyl	**34**	50
9	4-Iodoanisole	**35**	43
10	3-Iodoanisole	**36**<br>**36a**	64<br>17

Table S6. Pd(0)-catalyzed arylation of trioxadiamine **1** with various aryl iodides.

Entry	Aryl halide	$Pd(dba)_2$/BINAP, mol%	Product	Yield, %
1	4-Bromobenzonitrile	1/1.5	**27**	84
2	3-Bromobenzonitrile	1/1.5	**28**	55 [a)]
3	3-Bromobenzonitrile	4/4.5	**28**	94
4	2-Bromobenzonitrile	1/1.5	**29**	69 [a)]
5	2-Bromobenzonitrile	4/4.5	**29**	40
6	4-Bromoacetophenone	1/1.5	— [b)]	—
7	3-Bromoacetophenone	1/1.5	— [b)]	—
8	4-Bromobenzophenone	1/1.5	**32**	96
9	4-Bromobiphenyl	1/1.5	**33**	65
10	3-Bromobiphenyl	1/1.5	**34**	79
11	4-Bromoanisole	1/1.5	**35**<br>**35a**	41<br>6
12	3-Bromoanisole	1/1.5	**36**	73
13	3-Bromobenzophenone	1/1.5	**37**	82

[a)] Total conversion of NH_2 into NHAr is shown which was estimated from ^{1}H NMR spectrum of the reaction mixture; [b)] Starting compound was unstable under reaction conditions.

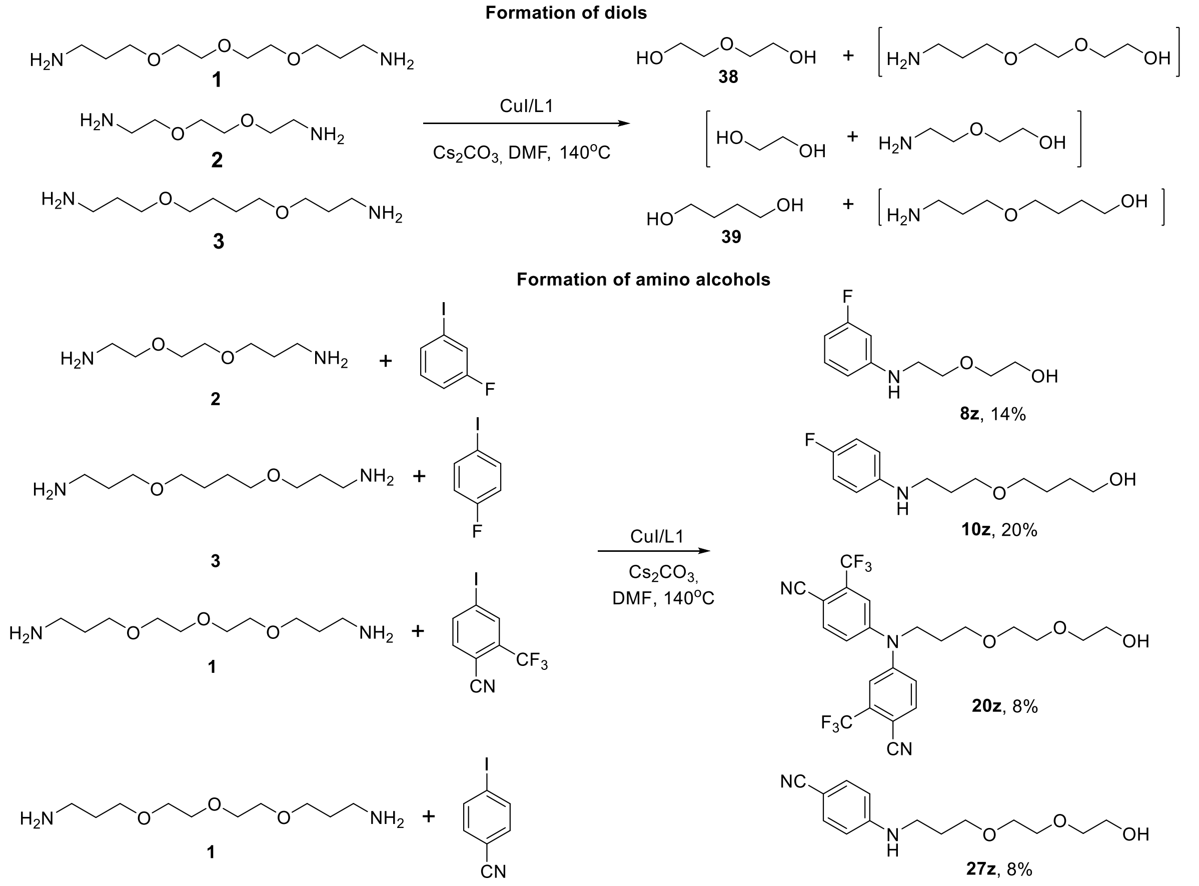

Scheme S5.

2-(2-((3-Fluorophenyl)amino)ethoxy)ethan-1-ol (**8z**). Obtained as the second product in the synthesis of compound **8**using method A. Eluent CH2Cl2–MeOH 50:1. Yield 14 mg (14%). ^{1}H-NMR (400 MHz, $CDCl_3$) δ 3.29 (t, 2H, ^3J = 5.6 Hz, CH_2N), 3.59 (t, 2H, $^3J_{HH}$= 4.5 Hz, OCH_2), 3.70 (t, 2H, ^3J = 5.3 Hz, OCH_2), 3.70 (t, 2H, ^3J = 5.3 Hz, OCH_2), 6.31 (dt, 1H, $^3J_{HF}$= 11.5 Hz, $^4J_{HH}$= 2.1 Hz, H2 (Ph)), 6.36–6.41 (m, 2H, H4, H6 (Ph)), 7.09 (td, 1H, $^3J_{HH}$= 8.0 Hz, $^4J_{HF}$= 6.8 Hz, H5 (Ph)). NH and OH were not unambiguously assigned. ^{13}C-NMR (100.6 MHz, $CDCl_3$) δ 43.5 (1C, CH_2N), 61.8 (1C, OCH_2), 69.4 (1C, OCH_2), 72.1 (1C, CH_2OH), 99.6 (d, 2C, $^2J_{CF}$ = 25.1 Hz, C2 (Ph)), 104.0 (d, 1C, $^2J_{CF}$ = 21.4 Hz, C4 (Ph)), 108.9 (1C, C6 (Ph)), 130.2 (d, 1C, $^3J_{CF}$ = 10.3 Hz, C5 (Ph)), 149.9 (d, 1C, $^3J_{CF}$ = 10.3 Hz, C1 (Ph)), 158.7 (d, 1C, $^1J_{CF}$ = 242.2 Hz, C3 (Ph)). MS (MALDI-TOF+): Calculated for $C_{10}H_{13}FNO_2$ [*M* – H_2+ H] 198.0930, found 198.0911.

4-(3-((4-Fluorophenyl)amino)propoxy)butan-1-ol (**10z**). Obtained as the second product in the synthesis of compound **10** using method A. Eluent CH2Cl2–MeOH 50:1. Yield 24 mg (20%). ^{1}H-NMR (400 MHz, $CDCl_3$) δ 1.64–1.70 (m, 4H, OCH_2**CH_2CH_2**CH_2OH), 1.89 (quintet, 2H, ^3J = 6.2 Hz, OCH_2C**H_2**CH_2N), 3.20 (t, 2H, ^3J = 6.5 Hz, CH_2N), 3.47 (t, 2H, ^3J = 5.7 Hz, OCH_2), 3.56 (t, 2H, ^3J = 5.8 Hz, OCH_2), 3.65 (t, 2H, ^3J = 5.7 Hz, CH_2O), 6.61 (dd, 2H, $^3J_{HH}$= 8.8 Hz, $^4J_{HF}$ = 4.3 Hz, H2, H2′ (Ph)), 7.85–7.89 (dd, 2H, $^3J_{HH}$= 8.8 Hz, $^3J_{HF}$ = 8.8 Hz, H3, H3′ (Ph)). ^{13}C-NMR (100.6 MHz, $CDCl_3$) δ 26.5 (1C, CH_2**C**$H_2CH_2CH_2OH$), 28.0 (1C, CH_2CH_2**C**H_2CH_2OH), 29.9 (1C, NCH_2**C**H_2CH_2O), 45.3 (1C, CH_2N), 62.5 (1C, CH_2O), 69.0 (1C, OCH_2), 71.1 (1C, OCH_2), 116.0 (d, 2C, $^2J_{CF}$= 22.5 Hz, C3, C3′ (Ph)), 117.1 (d, 2C, $^3J_{CF}$= 7.5 Hz, C2, C2′ (Ph)), 149.8 (1C, C1 (Ph)), 157.8 (d, 1C, $^1J_{CF}$= 240.5 Hz, C4 (Ph)). MS (MALDI-TOF+): Calculated for $C_{13}H_{21}FNO_2$ [*M* – H_2+ H] 242.156, found 242.173.

4-((3-(2-(2-Hydroxyethoxy)ethoxy)propyl)amino)-2-(trifluoromethyl)benzonitrile (**20z**). Obtained as one of several products according to method A from trioxadiamine **1** (0.5 mmol, 110 mg), 4-iodo-2-(trifluoromethyl)benzonitrile (2 mmol, 594 mg) in the presence of CuI (19 mg) and 2-isobutyrylcyclohexanone (34 mg). Eluent CH2Cl2–MeOH 100:1. Yield 20 mg (8%). ^{1}H-NMR (400 MHz, $CDCl_3$) δ 1.94 (quintet, 2H, ^3J = 6.2 Hz, OCH_2C**H_2**CH_2N), 3.52 (t, 2H, ^3J = 6.9 Hz, CH_2NPh_2),

3.59–3.63 (m, 4H, OCH_2), 3.66–3.79 (m, 2H, OCH_2), 3.74 (t, 2H, ^3J = 4.5 Hz, C**H**$_2$OH), 4.05 (t, 2H, ^3J = 6.9 Hz, CH_2NPh_2), 7.36 (dd, 4H, ^3J = 8.6 Hz, ^4J = 2.3 Hz, H6 (Ph)), 7.47 (d, 4H, ^4J = 2.3 Hz, H2(Ph)), 7.76 (d, 4H, ^3J = 8.6 Hz, H5(Ph)). OH proton was not unambiguously assigned. ^{13}C-NMR (100.6 MHz, $CDCl_3$) δ 27.2 (1C, OCH_2**C**H_2CH_2N), 49.2 (1C, CH_2N), 61.7 (1C, CH_2OH), 67.2 (1C, OCH_2), 70.3 (1C, OCH_2), 70.4 (1C, OCH_2), 72.3 (1C, CH_2O), 103.0 (2C, C4 (Ph)), 115.5 (2C, CN), 118.7 (2C, C2 (Ph)), 122.0 (q, 2C, $^1J_{CF}$ = 274.4 Hz, CF_3), 123.5 (2C, C6 (Ph)), 134.8 (q, 2C, $^2J_{CF}$ = 32.5 Hz, C3 (Ph)), 136.4 (2C, C5 (Ph)), 149.4 (2C, C1 (Ph)). ^{19}F-NMR (376.4 MHz, $CDCl_3$) δ –62.33 (6F, CF_3). MS (MALDI-TOF+): Calculated for $C_{23}H_{22}F_6N_3O_3$ [*M* + H] 502.1565, found 502.1530.

4-((3-(2-(2-Hydroxyethoxy)ethoxy)ethoxy)amino)benzonitrile (**27z**). Obtained as the second product in the synthesis of compound **27**. Eluent CH_2Cl_2/MeOH 20:1 Yield 11 mg (8%). ^{1}H-NMR (400 MHz, $CDCl_3$) δ 1.89 (quintet, 2H, ^3J = 5.9 Hz, OCH_2C**H**$_2CH_2N$), 3.26 (q, 2H, ^3J = 6.3 Hz CH_2N), 3.58–3.62 (m, 6H, OCH_2), 3.65–3.68 (m, 2H, OCH_2), 3.73 (t, 2H, ^3J = 4.4 Hz, CH_2O), 4.90 (br. s, 1H, NH), 6.53 (d, 2H, $^3J_{obs}$ = 8.7 Hz, H2, H2'(Ph)), 7.37 (d, 4H, $^3J_{obs}$ = 8.7 Hz, H3, H3'(Ph)). OH proton was not unambiguously assigned. MS (MALDI-TOF+): Calculated for $C_{14}H_{27}N_2O_3$ [*M* + H] 265.1552, found 265.1524.
